# Political Ideology Direction of Policy Agendas and Maternal Mortality Outcomes in the U.S., 1915–2007

**DOI:** 10.1007/s10995-023-03859-2

**Published:** 2024-01-02

**Authors:** Javier M. Rodriguez, Byengseon Bae

**Affiliations:** Department of Politics & Government, Claremont Graduate University, 150 E 10th St, Claremont, CA 91711 USA

**Keywords:** Maternal mortality rate, Political realignment, Parties, Conservatism, Social determinants of health

## Abstract

**Objectives:**

The causes for persistently high and increasing maternal mortality rates in the United States have been elusive.

**Methods:**

We use the shift in the ideological direction of the Republican and the Democratic parties in the 1960s, to test the hypothesis that fluctuations in overall and race-specific maternal mortality rates (MMR) follow the power shifts between the parties before and after the Political Realignment (PR) of the 1960s.

**Results:**

Using time-series data analysis methods, we find that, net of trend, overall and race-specific MMRs were higher under Democratic administrations than Republican ones before the PR (1915–1965)—i.e., when the Democratic Party was a protector of the Jim Crow system. This pattern, however, changed after the PR (1966–2007), with Republican administrations underperforming Democratic ones—i.e., during the period when the Republican Party shifted toward a more economically and socially conservative agenda. The pre-post PR partisan shifts in MMRs were larger for Black (9.5%, $p<.01$) relative to White mothers (7.4%, $p<.05$) during the study period.

**Conclusions for Practice:**

These findings imply that parties and the ideological direction of their agendas substantively affect the social determinants of maternal health and produce politized health outcomes.

**Supplementary Information:**

The online version contains supplementary material available at 10.1007/s10995-023-03859-2.

## Significance

**What Is Already Known on This Subject?:**

The causes for persistently high and increasing maternal mortality rates in the United States have been elusive.

**What This Study Adds?:**

We find that, net of trend, MMRs are higher under the most racially-conservative party of the period: The Democratic Party before the Political Realignment, the Republican Party after. Institutionalized racism—in the form of racialized federal-level policy—has detrimentally affected the health of all in the past century. Our findings bear important implications for political science, medical sociology, public health, and policy: A deep understanding of political processes is necessary for promoting health equity.

**Supplementary Information:**

The online version contains supplementary material available at 10.1007/s10995-023-03859-2.

## Objectives

**Fig. 1:**
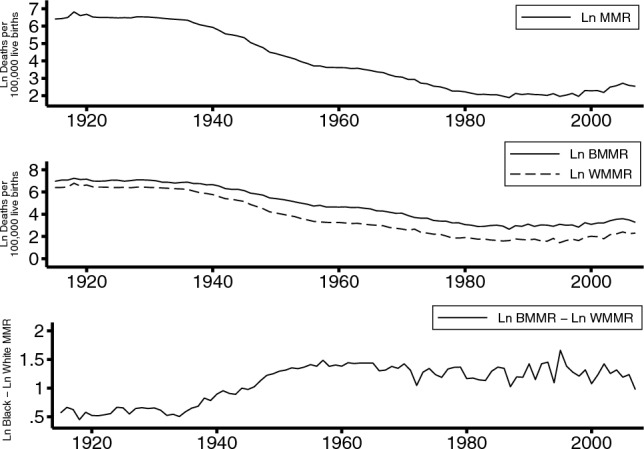
Time trends of overall and race-specific maternal mortality rates, and racial gaps and ratios. Ln MMR, natural logarithm of maternal mortality rate; Ln BMMR, natural logarithm of Black maternal mortality rate; Ln WMMR, natural logarithm of White maternal mortality rate

Pregnant women in the U.S. are exposed to a higher risk of death than those in other similarly developed countries. For instance, the U.S. maternal mortality rate (MMR) in 2017 was 19 maternal deaths per 100,00 live births and 7, 7, and 8 for the United Kingdom, Germany, and France respectively (WHO, 2019). Although MMRs decreased drastically in the U.S. during the twentieth century—from 607.9 in 1915 to 12.7 in 2007, stagnating in the 1980s and then increasing since the 1990s (Fig. 1)—the Black-to-White MMR ratio increased from 1.76 in 1915 to 2.65 in 2007, revealing that overall improvements in MMR were not necessarily translated into racial equity.

The causal factors underlying these trends have been elusive to policy-makers and researchers. While public health scientists have traditionally focused on biological causes such as cardiovascular disease and infections, healthcare institutional causes like access to and quality of services, and contextual conditions like socioeconomic status and pollution (Collier & Molina, 2019; Howell & Zeitlin, 2017), this study points to the hypothesis that the power vested in political institutions and the ideological direction of their policy agendas represent a part of the fundamental causal mechanism guiding overall and race-specific fluctuations in MMRs in the U.S. in the past century.

Research has effectively identified a set of proximal medical conditions related to maternal deaths—e.g., cardiomyopathy, infection or sepsis, hemorrhage, cerebrovascular accidents, hypertensive disorders of pregnancy, amniotic fluid embolism, and anesthesia complications during labor (CDC, 2022)—of which cardiovascular conditions account for about one-third of pregnancy-related deaths (Collier & Molina, 2019). Increasing maternal age, obesity, suicide, accidental drug overdose, and intimate partner violence are also commonly identified as other socio-behavioral proximal risk factors for maternal deaths (Collier & Molina, 2019; Hameed et al., 2015). Significant racial/ethnic disparities in MMRs have been attributed to differential access to obstetric care delivery and hospital quality of care (Howell & Zeitlin, 2017; Howell et al., 2017). Yet, research shows that such disparities persist even after controlling for patient- and hospital-level factors, suggesting that there are aspects of structural racism and institutional factors not captured by analyses merely focusing on established social determinants of health.

From a political epidemiologic perspective, we argue that the social determinants of health are largely affected by political forces (Barnish et al., 2018, 2021; Rodriguez, 2019). We observe that there is no social determinant of health that is not preceded by a set of policies and programs prescribed by politicians and that is at least initially provisioned by and executed through the governmental apparatus. Indeed, there is no health policy or program implementation (i.e., the immediate predecessors of the social determinants of health) not likewise preceded by underlying political institutions, the law, and the rules and regulations that structure governance operationally and administratively.

New growing evidence is uncovering a variety of connections between politics-specific factors and population health. From linking variations in life expectancy and racial differential mortality with electoral outcomes and political inequality (Bilal et al., 2018; Rodriguez et al., 2015) to connecting patterns in tax policy and females’ participation in legislatures with survivability and infant mortality rates (Homan, 2017; Kim, 2018), these emerging patterns strongly suggest that political apparatuses and power dynamics are factors that should be seriously considered fundamental causes of population health.

Although the presidency is not the only political institution affecting health policy in the U.S., there is good reason to think that presidents, the parties they represent, and their ideologies have the capacity to influence population health. In the U.S., presidents not only have the power to sign or veto legislation, command the armed forces, appoint and remove executive officers, and appoint judges. Presidents also exercise unilateral powers such as issuing executive orders, memoranda, proclamations, national security directives, and executive agreements (Relyea, 2007). Indeed, many of the presidential unilateral powers have been increasing in the last decades through massive appointments of presidential bureaucracies, staff, and gatekeepers that enable them to enact their agendas quite independently from Congress (Rodriguez, 2019).

Presidents also initiate policies and programs that affect the social determinants of health. Salient examples are the establishment of Social Security under Franklin D. Roosevelt and the series of policy and legislative reforms of the Great Society and the War on Poverty under Lindon B. Johnson—all of which continue to affect the health of all, today. The largest and most influential public health-specific programs—e.g., Medicaid, Medicare, CHIP—and reforms to them—e.g., the Affordable Care Act—have been introduced by presidents as well (Forgotson, 1967; Rodríguez et al., 2022), and have shown to deliver differential effects by race and other minority statuses (Ye & Rodriguez, 2021). There is also evidence that presidents and political processes explicit to the presidency—e.g., macroeconomic fluctuations during general elections—affect critical social determinants of health like unemployment, inflation, and income inequality (Bartels, 2016).

Progressive ideological movements across the globe tend to be associated with the enhancement of welfare regimes that in turn produce better health outcomes (Eikemo et al., 2008). In the U.S. context, evidence shows that, net of history, Republican presidential administrations—characterized by implementing more conservative policies especially after the 1960s—underperform Democratic ones in decreasing infant mortality rates, overall and by race (Rodríguez et al., 2022; Rodriguez et al., 2014a, 2014b). This pattern is also detected at the subnational level, with less conservative U.S. states manifesting better life expectancies and lower infant mortality rates than more conservative ones (Montez, 2020; Rodriguez et al., 2022).

These findings suggest that the ideological direction of policy (liberal/progressive vs. conservative) affects the social determinants of health. Liberal policies such as increasing the minimum wage and generous paid family-leave correlate with improved infant health in U.S. states (Komro et al., 2016; Stearns, 2015). Higher mortality is associated with lower expenditure in welfare programs and education—which have higher effects on mortality than investment in health-specific infrastructure like hospitals (Fenelon & Witko, 2021; Montez et al., 2016). Studies also suggest that political patterns are affected by health outcomes (Rodriguez et al., 2015)—e.g., counties that voted for Donald Trump in 2016 were showing lower gains in life expectancy, prolonged opioid prescriptions, and midlife mortality among non-Hispanic White Americans before the elections (Bilal et al., 2018; Goodwin et al., 2018).

Although the global initiative to reduce maternal mortality remains in its infancy, there is additional evidence that good governance—in terms of government effectiveness, regulatory quality, rule of law, control of corruption, voice and accountability, and political stability and absence of violence—reduces maternal mortality (Ruiz-Cantero et al., 2019). Indeed, the introduction of quotas for women in parliamentary democracies is associated with substantive declines in maternal mortality (Bhalotra et al., 2019). U.S. domestic presidential partisan politics affects maternal mortality in other countries, as international public aid for family planning is 48% higher under Democratic (relative to Republican) presidential regimes (Bhalotra et al., 2021). Except for a few recent studies conducted from a comparative perspective (Ruiz-Cantero et al., 2019), however, studies connecting politics to U.S. MMRs are scarce.

This study attempts to bridge this gap. By starting the clock in 1915, we use national-level data—the only format available for the study period—to investigate the relationship between the party that controls the presidency and MMRs in the U.S. Given the drastic shift in the ideological direction of national policy between the Republican and the Democratic parties during the Political Realignment (PR) of the 1960s, the PR represents a useful exogenous source of variation to test the hypothesis that MMR trends follow partisan power and political ideological dynamics in U.S. policy. In the 1960s, the Democratic Party shifted toward a less conservative (socially and racially) policy agenda. By abandoning its support for the Jim Crow system and by incorporating the Civil Rights Movement’s agenda, the Democratic Party contrasted the Republican Party which turned toward socioeconomic and racial conservatism. This dramatic transformation in the 1960s was crucial for population health, including infant mortality rates (IMR), which are related to maternal mortality (Forgotson, 1967; Rodríguez et al., 2022). In analyzing data after the PR, Rodriguez and colleagues show that, net of trend, IMR fluctuations follow the back-and-forth of presidential party regimes since 1965 (Rodriguez, 2019; Rodriguez et al., 2014a, 2014b; Rodriguez et al., 2022).

## Methods

Given that the PR transformed the ideology direction of the national policy agendas promoted by the political parties, it is expected that fluctuations in overall and race-specific maternal mortality rates (MMR) shift between the parties in power before and after the PR. Accordingly, we test the hypothesis that overall and race-specific MMRs were higher and lower under Democratic administrations than Republican ones *before* and *after* the PR, respectively—i.e., that MMRs are higher under the more conservative party of the time.

Data for MMRs (defined as the number of women deaths *that occur during pregnancy or within 42 days following the termination of the pregnancy per 100,000 live births)* are from the Vital and Health Statistics Series and from Linder and Grove (1943). Our data covers up to year 2007 since official data for MMRs after 2007 are unavailable. Other data capturing pregnancy-related deaths in the U.S. are much recent and do not cover the period of interest. The MMR data at subnational levels are highly limited. Given the length of our time series and the drastic changes that MMRs manifest from 1915 to 2007 (e.g., from a range of 608 to 916 (1915–1919) to a range of 12 to 15 (2002–2007)), we logged the MMR to stabilize its variance along the series.

The Black MMR data are for non-White women, 1915–1934, and for black women, 1935–2007. The bias due to a non-White categorization of the Black MMR data should be small as the great majority of the non-White population was Black in the early 1900s (e.g., 98% in 1900, and 91% in 1930). We also control for key confounders. The fertility rate data are from the National Vital Statistics Reports, the unemployment rate from the Bureau of Labor Statistics, the percentage of women aged 15–44 years from the Census Bureau, war years from Congressional Research Service, and recession years from the National Bureau of Economic Research. (Table A1 in the Supplementary Information lists our variables description.)

Our models are of the form:

1 $${M}_{t}={\beta }_{0}+{\beta }_{1}{R}_{t}+{\beta }_{2}{Post}_{t}+{\beta }_{3}{\left(Post*R\right)}_{t}+\sum_{i=1}^{n}{\delta }_{i}{X}_{it}+{\varepsilon }_{t}$$

where $M$ is the natural logarithm of the MMR in year *t*. Because presidential policy does not produce immediate effects (Rodriguez et al., 2014a) the term ${R}_{t}$ is a 1-year lagged dummy denoting if the president was a Republican (1 = Republican; 0 = Democrat). ${Post}_{t}$ stands for the post Political Realignment period (coded “0” for 1915–1965, “1” after 1965). Estimates of ${\beta }_{1}$ represent the net-of-trend logged-MMR differential of Republican versus Democratic administrations before the Political Realignment; estimates of ${\beta }_{3}$ represent the shift in this differential post Realignment, thus testing for the Republican-Democratic president difference in ${M}_{t}$ from before to after the Political Realignment. The post Realignment differential, itself, is ${\beta }_{1}+{\beta }_{3}$. The expression $\sum_{i=1}^{n}{\delta }_{i}{X}_{it}$ represents a vector of *n* controls ${X}_{it}$(i.e., war years, recession years, logged fertility rate, logged unemployment rate, and logged percentage of women aged 15–44) with their respective coefficients ${\delta }_{i}$. ${\varepsilon }_{t}$ is the regression error term.

All variables except for the president’s party, and war and recession years, were de-trended using the Hodrick-Prescott (HP) filter with smoothing parameter $\gamma =100$—a commonly-used tool for de-trending mortality data (Granados, 2005). We also experimented with other forms of de-trending the series described in Rodriguez et al., (2014a, 2014b) (e.g., using 8-, 9-, and 10-knot cubic splines (Tables A2 and A3, Supplementary Information)). The standard errors of the coefficients are estimated using the Newey-West estimator, which is robust to arbitrary patterns of serial correlation. To avoid underestimating sampling variability, we adjusted the 95% confidence intervals and the *p* values using fixed-b asymptotics (Vogelsang, 2012). The distributions of the errors ${\varepsilon }_{t}$ are assumed heteroskedastic and autocorrelated up to a lag of 3 years (Relyea, 2007; Rodriguez, 2019). We estimated Eq. 1 for overall MMR, and separately for Black and White MMRs.

This is an observational study that does not involve obtaining information about living individuals. This study is not based upon clinical study or patient data.

## Results

**Fig. 2:**
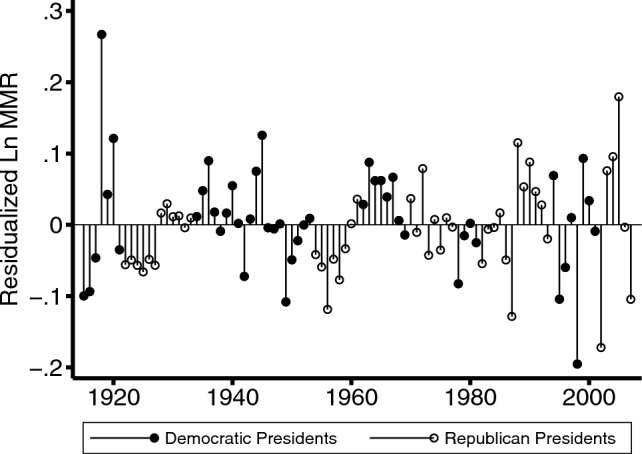
Unadjusted trend for residualized Ln MMR (Hodrick-Prescott filter (*γ* = 100)) by presidential party, 1915–2007. Ln MMR, natural logarithm of maternal mortality rate

Figure 1 shows that MMRs fell dramatically over the last century, with evidence of an actual increase in MMR over the last twenty-five years, overall and for Black and White women. In the bottom panel we show the racial gap in MMR. Distressingly, over the last half century, this gap has stagnated at high levels of MMRs. Figure 2 shows the unadjusted trend for residualized logged MMR by presidential party, 1915–2007.

Table 1 reports descriptive statistics for our de-trended variables, before and after the PR by presidential party. Our data cover the presidencies of 17 presidents (i.e., 37% of all presidents in U.S. history), with higher time-shares under Democratic presidents before the PR (61%) and more under Republicans after the PR (62%). All means of our de-trended dependent variables were higher under the more racially conservative party of the time—i.e., Democratic administrations before the PR and then under Republican ones after the PR. Though the unadjusted partisan differences for MMRs are not precisely estimated for the post-PR, the differences are sharper during the pre-PR period and precisely estimated (*p* < .05, Table 1).

**Table 1:** Descriptive statistics, before and after the Political Realignment by presidential party, 1915–2007

Variable (de-trended)	Pre-PR (1915–1965)		Post-PR (1966–2007)	
	Mean Dem	Mean Rep	Mean Dem	Mean Rep
Ln MMR	.019	− .030	− .012	.008
Ln WMMR	.018	− .030	− .007	.007
Ln BMMR	.018	− .025	− .019	.010
President’s party affiliation^a^	.608	.392	.381	.619
Ln unemployment rate	.014	.004	− .114	.050
Ln percentage of women aged 15–44	.001	− .003	.000	.000
Ln fertility rate	− .003	.009	−.008	.001
War year^a^	.516	.100	.250	.308
Recession year^a^	.290	.600	.188	.231

^a^Not de-trended. President’s party is 1-year lagged

To account for the fraction of the population at risk of pregnancy and for selective fertility, we controlled for the percentage of women aged 15–44 and for the fertility rate, with no meaningful differences in these indicators between the parties neither before nor after the PR. We also included two macroeconomic indicators, with more recession years under Republican administrations before and after the PR, and the unemployment rate being higher under the more conservative party both before and after the PR—i.e., higher under Democratic administrations before the PR and higher under Republican ones after the PR. This last pattern is also detectable for war years: higher under Democratic before the PR, and higher under Republican administrations after the PR.

Table 2 reports regression parameter estimates. The left panel is for models without controls, the right for models with controls. Interpreting models with controls, in all instances, MMRs showed to be lower under Republican presidents before the PR and then higher after the PR, net of trend. We also report the pre-post PR difference (i.e., estimates of ${\beta }_{3}).$ In all cases, our estimates of ${\beta }_{3}$ are precisely estimated (*p* < .05), large (all above a 7% pre-post partisan difference), and in the direction of our expectations (Figure A1 in the Supplementary Information visualizes our results).

**Table 2:** Parameter estimates for presidential party (Republican) effects on overall and race-specific maternal mortality rates before and after the Political Realignment, 1915–2007

	Without Controls (Model 1)			With Controls (Model 2)		
	Pre-PR	Post-PR	Difference	Pre-PR	Post-PR	Difference
Ln MMR						
Coefficient Estimate	− .049	.019	.068	− .059	.028	.086
95% Confidence Interval	± .041	± .043	± .058	± .041	± .041	± .060
*P*-value	.019	.374	.023	.006	.186	.005
Ln WMMR						
Coefficient Estimate	− .048	.014	.062	− .052	.021	.074
95% Confidence Interval	± .042	± .060	± .073	± .043	± .057	± .071
*P*-value	.026	.637	.092	.017	.451	.043
Ln BMMR						
Coefficient Estimate	− .042	.029	.071	− .058	.036	.095
95% Confidence Interval	± .039	± .045	± .059	± .046	± .048	± .069
*P*-value	.035	.208	.020	.015	.132	.008

Model 1 is without controls; model 2 controls for Ln unemployment rate, Ln percentage of women aged 15-44 years, Ln fertility rate, war year, and recession year

## Conclusions for Practice

This study found that, net of trend, overall and race-specific MMRs were higher under Democratic administrations relative to Republican ones before the PR, and higher under Republican administrations than under Democratic ones after the PR. Overall and race-specific MMRs were respectively higher under the more conservative party of the time. This suggests that population health in the U.S. is politically patterned by means of racialized policy (Michener, 2019; Rodríguez et al., 2022). We also found that the partisan shifts in MMRs from before to after the Political Realignment are large and precisely estimated, suggesting that population health in the U.S. is mutable according to political factors, and that the ideological direction of policy and the parties that promote such policies represent powerful determinants of maternal health.

These findings are consistent with the politics hypothesis, which states that the production and distribution of the social determinants of health that emanate from social policy are political constructions that reflect the ideological and institutional climates of the time (Rodriguez, 2019). There is a set of stable, long-lasting political structures preceding the policies behind the evolution of every social determinant of health. That MMR trends are racially patterned indicates that the racialization of policy conflates race and socioeconomic status and that in the process of detrimentally affecting the health of Black mothers, it also affects the health of other groups that share their socioeconomic and political standing—e.g., poor White mothers, and very probably Native Americans and Hispanic mothers.

Our estimates represent annual average partisan differences in MMRs, either period-specific or between pre- and post-PR periods. The parameter estimates in Table 2 show, net of trend, a decrease in the overall MMR of 5.9% under Republicans during the pre-PR period (i.e., the less racially conservative party of the time) and an increase of 2.8% under Republicans during the post-PR period (i.e., the more racially conservative party of the time)—for a total shift in the overall MMR of 8.6% from pre- to post-PR (*p* < .01).

Our findings suggest that partisan fluctuations in MMRs are not new: partisan politics have affected both White and Black mothers during the last century. The pre-post PR partisan shifts in MMRs were larger for Black (9.5%, *p* < .01) relative to White mothers (7.4%, *p* < .05). However, considering that Black mothers have died at more than double the rate of White mothers during the last century, our relative effects imply larger absolute detrimental effects for Black than White mothers, respectively, under the more conservative presidential regime of the time.

To put our parameter estimates into perspective, Nelson et al. (2018) estimated the effects of a series of key predictors of MMRs in the U.S. between 1997 and 2012. They find that 5.3% of the total MMR increase during their study period could be attributed to low education status—explicitly, the proportion of women of childbearing age not having completed high school/GED. Given that not attaining a high school degree or GED signals an increasingly smaller and vulnerable sector of the population across time (Bound et al., 2015), Nelson, Moniz, and Davis’s effect could be interpreted as an upper-bound effect of education on MMRs—i.e., on a population of mothers relatively more vulnerable than the one we analyze in our study period. Still, our annual average pre-post PR partisan shifts in MMRs were 79% and 40% larger for Black and White mothers, respectively, than the effects of education reported by Nelson et al.

Although MMRs have dramatically improved over the past century, our findings highlight that racial disparities in health are entrenched. There is no controversy: Having good population health is in the best interest of everyone. Thus, if racial disparities in health in general, and particularly in MMRs, are entrenched in spite of the advancement of technology, scientific discoveries, and effective policies and intervention designs, it is imperative to consider the possibility that resulting policies and public interventions have been racialized by the governmental apparatus, too. Structural and institutional racism should be acknowledged as part of our political system: promoting health equity obliges us, therefore, to identify how we can change U.S. politics and its racialized nature.

There is no particular reason why the scientific community must leave the final writing and application of scientifically-produced policies and programs mostly in the hands of politicians, governmental agencies, and their associates. A natural course of action is, therefore, to generate necessary legislation to guarantee operational, bureaucratic, and managerial independence from government of vital health institutions like the National Institutes of Health (NIH), the Health Resources and Services Administration (HRSA), the Centers for Disease Control and Prevention (CDC), and the Agency for Healthcare Research and Quality (AHQR) among others. And, in matters of social policy, scientists must be diverse and members and stakeholders from hard-hit communities must also be engaged.

There are some limitations to this study worth noting. It is expected that not all maternal deaths following a birth should be attributed to pregnancy. Conceptually, to the extent that maternal mortality reflects mothers’ health, we would expect that maternal mortality would also reflect cumulative factors that may have been removed after de-trending the data, and so would be less responsive to short term changes in the political environment. This observation also raises the possibility that our estimates would be biased due to omitted variables not accounted for. However, much of these factors would be better conceptualized as mediators, not confounders of the relationship between partisan control of the presidency—before and after the PR—and MMRs. Aside from the fact that the exogenous causes of partisan cycles are still elusive in political science research, our estimates were robust to controlling for key sociodemographic and macroeconomic indicators, and various de-trending strategies. And finally, our data series do not cover the most recent years of MMR history. Having more recent data would have added to the generalizability of our findings. However, recent studies looking at infant mortality rates and presidents show that long-term patterns are generalizable to recent presidencies (Rodriguez et al. 2022; Torche & Rauf, 2021). These studies indicate that there is no strong theoretical reason to assume that the long-term relationships between political institutions such as parties, the ideological orientation of the policies they enact, and health outcomes would significantly diverge in more recent years.

### Supplementary Information

Below is the link to the electronic supplementary material.

Supplementary file1 (PDF 264 KB)

## Data Availability

Available upon request.

## Code Availability

Available upon request.
