# Supplementary material for: Political Ideology Direction of Policy Agendas and Maternal Mortality Outcomes in the U.S., 1915–2007
Source: Matern Child Health J. 2024 Jan 2;28(5):865–72. doi: 10.1007/s10995-023-03859-2 (PMC11001747; doi:10.1007/s10995-023-03859-2)
Supplement: Supplementary file 1 — Supplementary file1 (PDF 264 KB) [file 10995_2023_3859_MOESM1_ESM.pdf]

Electronic Supplementary Information

Article title: Political Ideology Direction of Policy Agendas and Maternal Mortality Outcomes in the U.S., 1915-2007
Journal name: Maternal and Child Health Journal
Author names: Javier M. Rodriguez and Byengseon Bae
Affiliation: Claremont Graduate University
E-mail address of the corresponding author: byengseon.bae@cgu.edu

This document contains:

1. Tables

- Table A1. Variable definitions and sources.
- Table A2. Robustness checks: Parameter estimates for presidential party (Republican) effects on overall and race-specific maternal mortality rates before and after the Political Realignment, 1915-2007, using different de-trending strategies (HP-filter (γ=100), and 8-, 9-, and 10-knot cubic splines).
- Table A3. Robustness checks goodness of fit statistics: Sum of squared residuals for de-trended Ln MMRs using different de-trending strategies.

2. Figures

- Figure A1. Predicted values of overall and race-specific maternal mortality rates by presidential party before and after the Political Realignment, 1915-2007.

3. References

Tables

Table A1. Variable definitions and sources

Variable	Definition	Source
MMR	• 1915-1978: The number of women deaths that occur during pregnancy or within 1 year of termination of pregnancy per 100,000 live births<br>• 1979-2007: The number of women deaths that occur during pregnancy or within 42 days of termination of pregnancy per 100,000 live births	• 1915-2002 (All races and White): Hoyert (2007)<br>• 1915-1934 (Non-White): Linder and Grove (1943) [a]<br>• 1935-2002 (Black): Hoyert (2007)<br>• 2003-2007 (All races, White, and Black): National Vital Statistics Reports
President's party affiliation	A year in which a Republican president is in office.	
Unemployment rate	The number of unemployed people as a percentage of the labor force [b]	• 1915-1970: (Census1975)<br>• 1971-2017: US Bureau of Labor Statistics
Percentage of women aged 15-44	Percentage of women aged 15-44 years	• 1915-1989: Census<br>• 1990-2017: CDC
Fertility rate	Births per 1,000 women aged 15–44 years	• 1915-1970: (Census1975)<br>• 1971-2000: CDC<br>• 2001-2017: National Vital Statistics Reports
War year	A year in which official war lasts at least for one month	1915-2017: (CRS2019)
Recession year	A year in which economic contraction lasts at least for a quarter	1915-2017: NBER (National Bureau of Economic Research)

[a] Prior to 1933, data for birth-registration states only.

[b] Labor force data are restricted to people 14 years of age and older for the period 1915-1946, and restricted to people 16 years of age and older for 1947-2017.

Table A2. Robustness checks: Parameter estimates for presidential party (Republican) effects on overall and race-specific maternal mortality rates before and after the Political Realignment, 1915-2007, using different de-trending strategies (HP-filter (γ=100), and 8-, 9-, and 10-knot cubic splines)

	HP-filter			8-knot spline			9-knot spline			10-knot spline		
	Pre-PR	Post-PR	Difference	Pre-PR	Post-PR	Difference	Pre-PR	Post-PR	Difference	Pre-PR	Post-PR	Difference
**Ln MMR**												
Coefficient Estimate	−.059	.028	.086	−.039	.030	.069	−.031	.025	.056	−.009	.048	.056
95% Confidence Interval	±.041	±.041	±.060	±.043	±.050	±.066	±.047	±.044	±.065	±.027	±.044	±.050
P-value	.006	.186	.005	.079	.230	.041	.198	.251	.090	.510	.036	.028
**Ln WMMR**												
Coefficient Estimate	−.052	.021	.074	−.034	.025	.059	−.025	.020	.046	−.004	.040	.044
95% Confidence Interval	±.043	±.057	±.071	±.045	±.064	±.077	±.050	±.057	±.075	±.027	±.060	±.063
P-value	.017	.451	.043	.137	.429	.130	.307	.468	.224	.753	.189	.165
**Ln BMMR**												
Coefficient Estimate	−.058	.036	.095	−.033	.046	.079	−.030	.041	.071	−.013	.059	.071
95% Confidence Interval	±.046	±.048	±.069	±.037	±.054	±.064	±.045	±.055	±.070	±.032	±.051	±.059
P-value	.015	.132	.008	.079	.091	.015	.182	.143	.046	.422	.026	.019

Note: All models control for Ln unemployment rate, Ln percentage of women aged 15-44, Ln fertility rate, war year, and recession year.

Table A3. Robustness checks goodness of fit statistics: Sum of squared residuals for de-trended Ln IMRs and Ln MMRs using different de-trending strategies

	HP-filter	8-knot spline	9-knot spline	10-knot spline
Ln MMR	.4613	.4965	.5281	.4021
Ln WMMR	.6068	.6501	.7011	.5432
Ln BMMR	.6082	.6332	.7317	.5957

Figures

Fig.A1 Predicted values of overall and race-specific maternal mortality rates by presidential party before and after the Political Realignment, 1915-2007

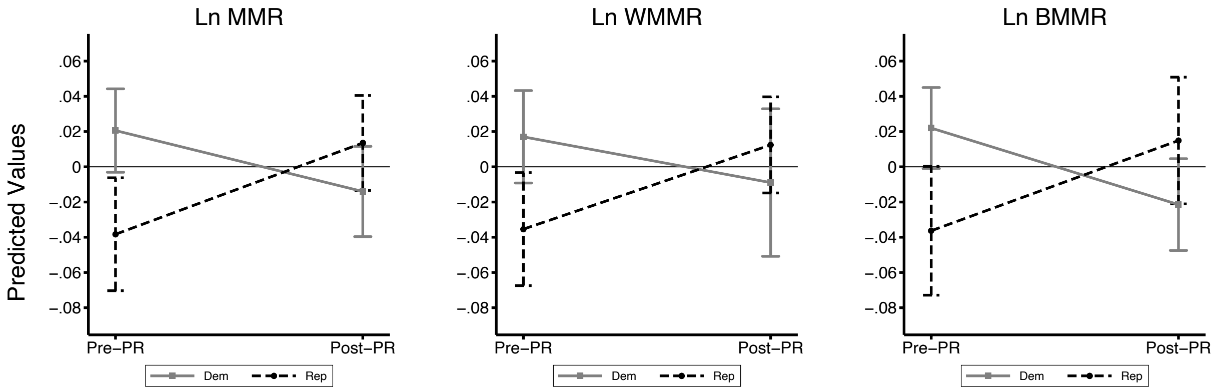

Note: The 95% CIs of predicted values are from standard errors calculated using the delta method.

References

Census (U.S. Bureau of the Census). (1975). *Historical Statistics of the United States, Colonial Times to 1970*. U.S. Govt. Print. Off.

CRS (Congressional Research Service). (2019). U.S. Periods of War and Dates of Recent Conflicts. *Congressional Research Service Report RS21405*.

Hoyert, D. L. (2007, Feb). Maternal Mortality and Related Concepts. *Vital Health Stat 3*(33), 1-13.

Linder, F. E., & Grove, R. D. (1943). *Vital Statistics Rates in the United States, 1900-1940*. U.S. Govt. Print. Off.

NBER (National Bureau of Economic Research). *US Business Cycle Expansions and Contractions*. Retrieved Jan 18, 2021 from https://www.nber.org/research/data/us-business-cycle-expansions-and-contractions
